# Production of Nano Hydroxyapatite and Mg-Whitlockite from Biowaste-Derived products via Continuous Flow Hydrothermal Synthesis: A Step towards Circular Economy

**DOI:** 10.3390/ma16062138

**Published:** 2023-03-07

**Authors:** Farah Nigar, Amy-Louise Johnston, Jacob Smith, William Oakley, Md Towhidul Islam, Reda Felfel, David Grant, Edward Lester, Ifty Ahmed

**Affiliations:** 1 Advanced Materials Research Group, Faculty of Engineering, University of Nottingham, Nottingham NG7 2RD, UK; 2 Bangladesh Council of Scientific and Industrial Research (BCSIR), Dhaka 1205, Bangladesh; 3 Food Water Waste Research Group, Faculty of Engineering, University of Nottingham, Nottingham NG7 2RD, UK; 4 School of Physical Sciences, University of Kent, Canterbury CT2 7NZ, UK; 5 Department of Applied Chemistry and Chemical Engineering, Faculty of Engineering, Noakhali Science and Technology University, Noakhali 3814, Bangladesh; 6 Department of Mechanical and Aerospace Engineering, Faculty of Engineering, University of Strathclyde, Glasgow G1 1XJ, UK; 7 Physics Department, Faculty of Science, Mansoura University, Mansoura 35516, Egypt

**Keywords:** biowastes, biowaste utilisation, continuous flow hydrothermal synthesis, sustainability, calcium phosphates, hydroxyapatite, Mg-whitlockite, eggshells, struvite

## Abstract

Biowastes from agriculture, sewage, household wastes, and industries comprise promising resources to produce biomaterials while reducing adverse environmental effects. This study focused on utilising waste-derived materials (i.e., eggshells as a calcium source, struvite as a phosphate source, and CH_3_COOH as dissolution media) to produce value-added products (i.e., calcium phosphates (CaPs) derived from biomaterials) using a continuous flow hydrothermal synthesis route. The prepared materials were characterised via XRD, FEG-SEM, EDX, FTIR, and TEM analysis. Magnesium whitlockite (Mg-WH) and hydroxyapatite (HA) were produced by single-phase or biphasic CaPs by reacting struvite with either calcium nitrate tetrahydrate or an eggshell solution at 200 °C and 350 °C. Rhombohedral-shaped Mg-WH (23–720 nm) along with tube (50–290 nm diameter, 20–71 nm thickness) and/or ellipsoidal morphologies of HA (273–522 nm width) were observed at 350 °C using HNO_3_ or CH_3_COOH to prepare the eggshell and struvite solutions, and NH_4_OH was used as the pH buffer. The Ca/P (atomic%) ratios obtained ranged between 1.3 and 1.7, indicating the formation of Mg-WH and HA. This study showed that eggshells and struvite usage, along with CH_3_COOH, are promising resources as potential sustainable precursors and dissolution media, respectively, to produce CaPs with varying morphologies.

## 1. Introduction

Developing sustainable materials and moving towards a circular economy are currently a focus for all major industries across the world. The use of waste materials as a resource for renewable raw materials could be utilised to address this issue [1,2,3,4,5].

Millions of tonnes of biowaste are produced annually worldwide from agriculture, sewage, households, food processing industries, and restaurants [6,7]. The disposal of wastes into water bodies and landfills creates concerns for waste management as well as environmental sustainability and consequently significantly threatens human and animal health [6,8]. On the other hand, these wastes are affluent sources of proteins (collagens, gelatines, eggshell membrane proteins, keratin, albumin, glycinin, etc.) [6,9,10], polysaccharides (cellulose, chitin, hyaluronic acid, pectin, etc.) [6,11,12,13] and bioceramics (hydroxyapatites, other calcium phosphates, larnite, wollastonite, etc.) [14,15]. In this context, the valorisation of wastes is of utmost importance because this potential alternative approach will reduce waste disposal and use natural resources for new materials development, which will increase the economic value of products. Some of these wastes could be utilised for the development of biomaterials such as calcium phosphates (CaPs) [16,17,18,19,20].

Calcium phosphates (CaPs) are the main mineral constituent of bones and teeth and possess excellent biocompatibility, osteoconductive properties, nontoxicity, and chemical similarity to the inorganic components of natural bone [21,22,23,24]. CaP-containing biomaterials are suitable for bone regeneration and replacement in tissue engineering as well as other applications, such as drug delivery agents, implant coatings, and gene carriers [23,25,26,27,28,29]

Among the CaPs compounds, hydroxyapatite Ca_5_(PO_4_)_3_OH (HA) is the most extensively used bone graft substitute due to its chemical and physical resemblance to the mineral part of bone tissue [30,31,32]. Biological HA is a nonstoichiometric, carbonated, and calcium-deficient form of apatite (with Ca/P ratio < 1.67), containing various amounts of positively charged ions (e.g., Mg^2+^, Na^+^, and K^+^) and negatively charged ions (e.g., CO_3_^2−^, Cl^−^, and F^−^), in substitution of Ca^2+^ or PO_4_^3−^/OH^−^ ions, respectively [21]. The second most abundant bone mineral in the human body is magnesium whitlockite (Mg-WH: Ca_18_Mg_2_(HPO_4_)_2_(PO_4_)_12_) which accounts for approximately 25–35 wt% of the inorganic part of human bone [33,34]. It is composed of a rhombohedral crystal structure with a cuboid shape [33]. In Mg-WH, magnesium is partly substituted for calcium [33,35] with a Ca/P ratio of 1.43 and is highly stable in acidic conditions [33]. Recent research on Mg-WH in the field of bone regeneration reported that this compound provided higher compressive strength than HA and had the ability to promote osteogenic activities simultaneously [36], thus promoting early-stage bone regeneration [34].

Several methods for synthesising nanocrystalline HA as well as mixed phases of CaPs have been developed, which include the in situ biomimetic process [37], hydrothermal synthesis [38], sol–gel precipitation [39], ultrasonic atomisation [40], ultrasonic coupled sol–gel synthesis [41] and wet ball-milling followed by sintering [42,43]. Among these processes, continuous flow hydrothermal synthesis appeared to be a resourceful method that could meet the growing demand for industrially sustainable routes to produce nanomaterials with varying morphologies [44,45].

Over the last few decades, scientists have investigated some biowastes, such as bovine bones, fish bones, avian eggshells, cuttlefish shells, oyster shells, and corals to develop calcium phosphates (CaPs) for biomedical applications [46,47,48,49,50,51,52,53,54,55] as they exhibit several advantages over synthetically developed materials in terms of biocompatibility, biodegradability, and remodelling [56,57]. For example, Kim et al. [57] prepared HA granules from cuttlefish bone (CB) via the hydrothermal method, and they reported that the CB-HA showed non-cytotoxicity, enhanced cell adhesion, proliferation, and differentiation (confirmed via in vitro and in vivo bioactivity tests), and higher bone growth during in vivo bone defect healing experiments. However, an assessment on the availability, ecological aspects, and ethical issues of these natural raw materials are important to consider for successful sustainable development [58,59,60]. Some of these materials may also contaminate the newly developed materials with toxic ions and consequently cause immunological responses in humans [57,60]. Considering these issues, eggshells as a potential source of calcium (which also have some natural trace elements such as K, Na, Mg, Si, and Sr) were selected to synthesise CaPs as they are abundantly available, have no ecological imbalance issues, and do not have associated ethical concerns [61,62].

Some common synthetic chemicals, e.g., diammonium hydrogen phosphate ((NH_4_)_2_HPO_4_) [44,63] and orthophosphoric acid (H_3_PO_4_) [64,65], have been used as phosphate precursors for CaPs preparation and are generally produced from naturally occurring phosphate rocks, which are a non-renewable mineral resource [66]. These natural P reserves are continuously depleted [67,68,69]. Bing et al. reported that phosphate rocks will be completely depleted within the next 70–140 years due to a lack of proper management [70]. For sustainable development, exploring alternative resources for phosphorus is essential. Therefore, phosphorus recovery from waste materials could be another promising approach in this regard [71]. Nowadays, effective and efficient recovery of P from non-conventional P-rich sources such as wastes and residues is gaining growing attention [67,72]. Various waste materials such as animal manures, municipal wastewater and biosolids, agricultural, and industrial effluents (i.e., production of chemicals, food and beverages, pharmaceuticals, tannery, textiles, semiconductor, potato processing, and molasses based food processing) [67] are enriched with higher nutrient content such as nitrogen (N) and phosphorous (P) [71] which are produced in large amounts every year globally [72]. On the other hand, the presence of P in wastewater is a key factor in water eutrophication [69]. Thus, non-treated wastewater does not only lead to water pollution but also contributes to the loss of potentially useful resources such as N and P. Therefore, recovering phosphorus from wastewater streams would be beneficial for waste management strategies, which could potentially be done through the precipitation of struvite (magnesium ammonium phosphate; MAP/MgNH_4_PO_4_ 6H_2_O) [67,69,71,73]. Struvite contains 39% phosphate [74] and thus has great potential for use as a natural P source [75].

Recently, researchers have been interested in studying waste-derived P sources (i.e., struvite from wastewater) [76,77,78], especially for agricultural applications. In a recent study, synthetic urine was chosen as a P source to investigate the synthesis of HA biomaterials entirely from biowastes [79]. Phosphorous (P) recovery from P-rich wastewater [80,81,82] through struvite formation [73] could be a useful resource for both environmental safety and nutrient recycling [71]. As such, struvite would be a potential P source for sustainable preparation of HA, addressing the circular economy. In addition, utilisation of acetic acid—produced from food waste and industrial wastewater streams—could be an another aspect towards sustainability and the circular economy for the manufacture of CaPs [83,84,85].

The hypothesis of this research was that nanocrystalline-CaP-based biomaterials could be manufactured from entirely waste-derived materials with minimal usage of synthetic chemicals. This study focused on the utilisation of waste-derived materials, eggshells, and struvite as precursors with acetic acid as the dissolution media to develop CaPs using a continuous flow hydrothermal synthesis process. To the best of our knowledge, no studies have reported on struvite as a P source to produce HA or CaPs for applications in the biomedical field. Initial trials used a conventional Ca source (i.e., calcium nitrate tetrahydrate solution) and replaced the conventional P source with struvite, used as an alternate potentially sustainable P source. In the second experimental trial, both Ca and P sources were replaced by waste-derived sources (i.e., eggshells and struvite) for exploring the formation of CaPs. In addition, the waste-derived precursor solutions were first prepared using nitric acid and then later acetic acid, which can also be prepared from food waste and potentially function as a sustainable dissolution media. Samples were also produced with varying pH buffers (NH_4_OH and NaOH) and temperatures (200 and 350 °C) to investigate the influence of these parameters on the morphology of CaPs produced. The materials produced were characterised using XRD, SEM, EDX, FTIR, and TEM analyses.

## 2. Materials and Methodologies

### 2.1. Materials

In this study, the precursors were calcium nitrate tetrahydrate (Ca(NO_3_)_2_.4H_2_O), eggshells and struvite (MgNH_4_PO_4_.6H_2_O). Nitric acid (HNO_3_) and acetic acid (CH_3_COOH) were used to dissolve waste-derived precursors (eggshells and struvite). Ammonium hydroxide (NH_4_OH) and sodium hydroxide (NaOH) were used as pH buffers. Eggshells were collected from household wastes, struvite was purchased from Alfa Aesar (UK), and the other chemicals used were obtained from Merck Life Science UK Limited.

### 2.2. Methodologies

#### 2.2.1. Calcium Nitrate Tetrahydrate (Ca(NO_3_)_2_.4H_2_O) Solution

A solution of 0.05 M calcium nitrate tetrahydrate was prepared by dissolving it in deionised water. The pH of the solution was maintained at 11 using NH_4_OH and NaOH.

#### 2.2.2. Struvite (MgNH_4_PO_4_.6H_2_O) Solution

To prepare a 0.015 M solution, struvite was dissolved in deionised water, followed by a dropwise addition of conc. HNO_3_ with continuous stirring until a clear solution was obtained. The pH of these solutions was maintained at 6 using NH_4_OH and NaOH individually. In addition, a second batch of struvite solutions was prepared by dissolving it in acetic acid (struvite:acetic acid = 1:3) instead of nitric acid, we followed the same procedure mentioned above for nitric acid. Only NH_4_OH was used to adjust the pH.

#### 2.2.3. Eggshell Solution Preparation

The eggshells collected were pre-treated by first removing the membranes manually and then boiling them in water at 100 °C for 30 min. After drying them overnight, eggshells were ground using a ball mill at 500 rpm for 10 min. After grinding, the eggshell powder was sieved to less than 45 µm to get a uniform fine powder.

To prepare a 0.05 M eggshell solution, ground eggshell powder (<45 µm) was dissolved in conc. HNO_3_ with a ratio of 1:2.5 (*w*/*v*). The undissolved eggshell substances were removed by filtration. This solution was then used as a stock solution for further experiments.

CaCO3 + 2 HNO3 = Ca(NO3)2 + H2O + CO2(1)

Eggshell solutions of pH 11 were prepared using two different base solutions, namely NH_4_OH and NaOH.

Another batch of eggshell solutions of the same concentration and volume was prepared, maintaining the same protocol, using acetic acid (CH_3_COOH) instead of HNO_3_ to dissolve the eggshell powder with a ratio of 1:3.63 (*w*/*v*) and NH_4_OH was used to adjust the pH of the solution.

CaCO_3_ + 2 CH_3_COOH = Ca(CH_3_COO)_2_ + H_2_O + CO_2_ (2)

#### 2.2.4. Hydrothermal Synthesis Process

Samples were prepared via a continuous flow hydrothermal synthesis route using a symmetrical counter-current nozzle reactor; the detailed method is described elsewhere [44]. Briefly, calcium solution (calcium nitrate/eggshell solution) was pumped (10 mL/min) into the reactor as up-flow, and simultaneously, 0.015 M struvite solution was introduced as the down-flow solution in the system, which was pumped (at 20 mL/min) through a preheater to the reactor chamber. The temperature was maintained at 200 °C and 350 °C (as highlighted in the sample codes), which were measured using a thermocouple, and the pressure was kept at 240 bar with the use of a back-pressure regulator.

#### 2.2.5. Sample Preparation Using Ca(NO_3_)_2_.4H_2_O as Ca and Struvite as P Sources

Five samples were prepared at 200 and 350 °C using struvite solution with Ca(NO_3_)_2_.4H_2_O as presented in Table 1; we followed the same procedures for solution flow rate and reactor pressure as mentioned above.

#### 2.2.6. Sample Preparation Using Eggshells as Ca and Struvite as P Sources

Five samples were also prepared at 200 and 350 °C using struvite solution and eggshell solution, as presented in Table 2, we followed the same procedures for solution flow rate and reactor pressure as mentioned above.

## 3. Characterisation Techniques

### 3.1. Elemental Analysis of Eggshells Via ICP-MS

Compositional analysis of the eggshell powder was carried out using inductively coupled plasma mass spectroscopy (ICP-MS, Thermo-Fisher Scientific iCAP-Q equipped with collision cell technology with energy discrimination) to verify their composition. A 0.1 g amount of eggshells were digested in 50 mL of 37% HCl until a clear solution was obtained. The solution was then 50% diluted with Milli-Q water (1:1). The resultant solution was then diluted with 2% HNO_3_ in a 1:10 ratio. Then the final solution was filtered through a 0.2 µm syringe filter for ICPMS analysis. Additionally, using a standard calibration solution, a blank solution was prepared following the same procedure. Three replicates were analysed, and the average values were reported.

### 3.2. X-Ray Diffraction (XRD)

X-ray diffraction studies were conducted to confirm the phase identification of the samples produced using a Bruker D8 Advance with a DaVinci diffractometer. The instrument was operated at room temperature and ambient atmosphere with Ni-filtered CuKα radiation (λ = 0.15418 nm), generated at 40 kV and 35 mA. Scans were performed with a step size of 0.01° and a step time of 2 s over an angular range 2*θ* from 5° to 65°. Diffraction patterns were analysed using EVA processing software for phase identification.

The average crystallite size of the synthesised particles was also calculated using Scherrer’s formula in Equation (3) [61], as follows:

(3) $$\mathrm{Xs}=\frac{0.9\lambda }{FWHMcos\theta }$$

where:

X_s_ = The average crystallite size (nm);

*λ* = The wavelength of the X-ray;

FWHM = The full width at half maximum for the diffraction peak under consideration (rad);

*θ* (degree) = The Bragg angle.

The percentage of phases produced in biphasic CaPs were measured according to Equation (4) [86] as follows:

(4) $$\mathrm{Percentage}\;\mathrm{of}\;\mathrm{HA}=\frac{I_{HA\left(211\right)}}{I_{HA\left(211\right)}+I_{Mg-WH\left(0210\right)}}$$

where:

*I_HA_*_(0210)_ = Intensity of HA at (211);

*I_Mg-WH_*_(0210)_ = Intensity of Mg-WH at (0210).

The crystallinity index of the phases in the samples was calculated from the FWHM of the 002 reflection of HA and the 0210 reflection of Mg-WH using Equation (5) [86].

(5) $$\mathrm{Crystallinity}\;\mathrm{index},\;\mathrm{CI}\;={\left(\frac{0.24}{\beta 002}\right)}^{3}$$

where:

*β*002 = full width at half maximum (FWHM) at (002) reflection of HA.

### 3.3. Scanning Electron Microscopy (SEM) and Energy Dispersive X-Ray Analysis (EDX)

Sample morphology was examined using Field Emission Gun Scanning Electron Microscopy (JEOL 7100F FEG-SEM), while an energy dispersive X-ray spectrometer (EDX) was used to determine the composition. An accelerating voltage of 5 kV was used for morphological analysis of the samples obtained.

EDX was conducted using an Oxford Instruments AZtec Energy Advanced X-max 150 EDS System for the chemical characterisation. A working distance of 10 mm was maintained for both SEM and EDX analysis. Samples were coated with iridium (Ir) using a Quorum Iridium coater for acquiring good-quality images via FEG-SEM. Carbon coating was done using Quroum Q150 for elemental analysis, and EDX spectroscopy was conducted using a working distance of 10 mm and a beam voltage of 15 kV.

### 3.4. Transmission Electron Microscopy (TEM)

TEM was conducted using a high-resolution TEM JEOL 2000fx at an accelerating voltage of 200 kV. The products, in powder form, were suspended in distilled water and dropped onto copper grids (300 lines per inch) prior to drying under vacuum for 15 min.

### 3.5. Particle Size Analysis

Particle size analysis was carried out using ImageJ software from the FEG-SEM images. For this measurement, 20 particles were considered.

### 3.6. FTIR Analysis

Infrared spectroscopy was performed using a Bruker Tensor-27 spectrometer (Germany). All samples were scanned in transmittance mode in the region of 4000 to 550 cm^−1^ (wave numbers) and the spectra were collected with a resolution of 4 cm^−1^ by averaging 32 scans using a standard Pike attenuated total reflectance (ATR) cell (Pike technology, UK). All spectra obtained were analysed using Opus^TM^ software version 5.5.

## 4. Results

### 4.1. Elemental Analysis

Table 3 represents the mol% and wt% composition of the eggshells collected, which were found to contain around 98 wt% of Ca with traces of other elements.

### 4.2. Hydroxyapatite Synthesis Using Calcium Nitrate Tetra-Hydrate—Struvite

#### 4.2.1. Investigating Phase Changes Using XRD

**Figure 1:**
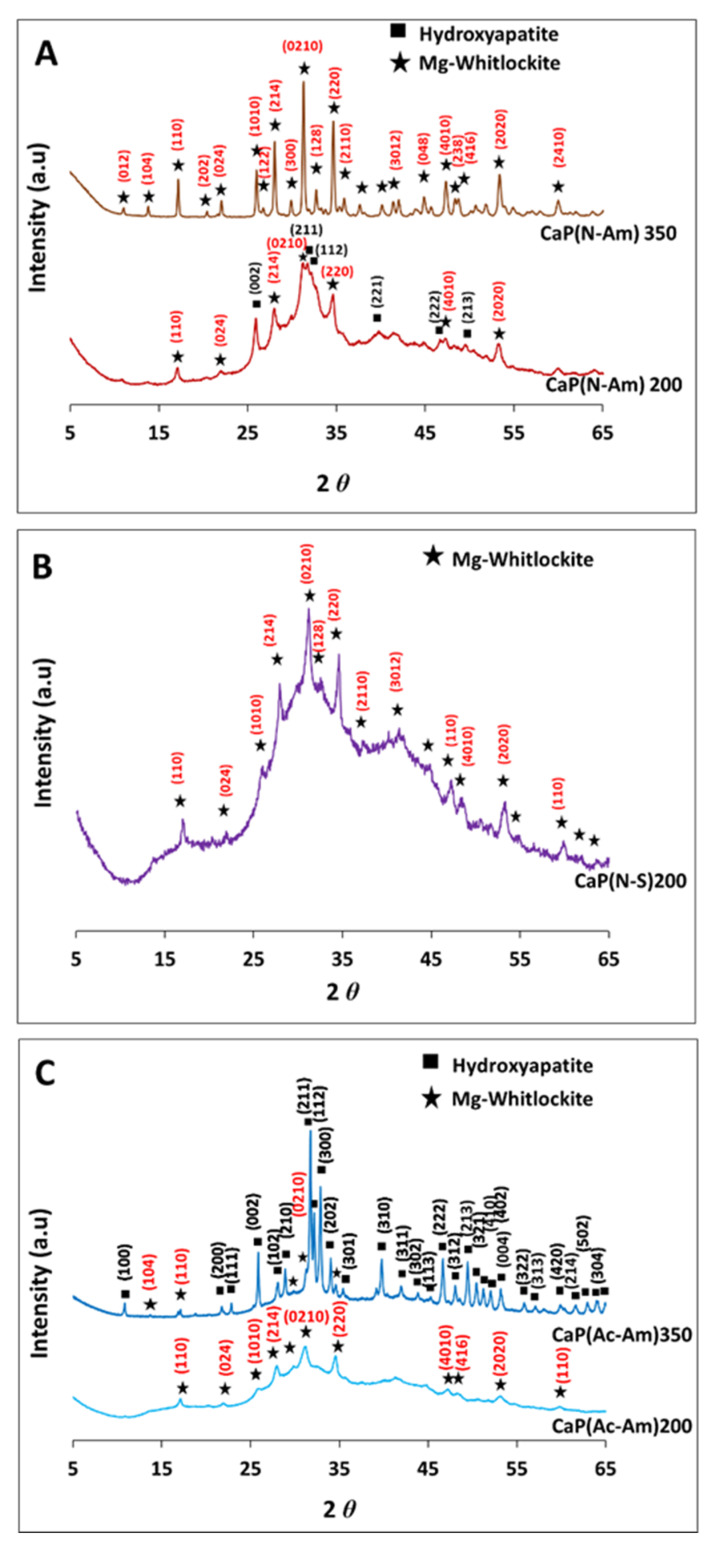
The XRD spectra of samples synthesised at 200 °C and 350 °C via a hydrothermal synthesis process at constant pH 11 of calcium nitrate tetrahydrate solution and pH 6 of struvite solution adjusted using (**A**,**C**) NH_4_OH (Am) and (**B**) NaOH (S), individually where struvite was dissolved in (**A**,**B**): HNO_3_ (N) and (**C**) CH_3_COOH (Ac). Calcium nitrate tetrahydrate was dissolved in DI water (W).

Figure 1 represents the XRD spectra for the samples obtained from the initial trials of three sets of experiments at 200 °C and 350 °C, using struvite and calcium nitrate tetrahydrate solutions of pH 6 and 11, respectively. Figure 1A,B show the effect of the two base solutions, i.e., NH_4_OH and NaOH (which were used to adjust the pH of the precursors), on the products formed, where struvite was dissolved in HNO_3_. A mixture of HA and mg-whitlockite (Mg-WH) formed at 200 °C, whereas a single phase of Mg-WH was obtained at 350 °C when NH_4_OH was used to adjust the pH (Figure 1A). However, semicrystalline Mg-WH had formed at 200 °C when using NaOH as a base solution. Interestingly, solid particles obtained at 350 °C dissolved in the product solution in a few minutes (Figure 1B). Figure 1C shows the XRD spectra of the samples produced at both temperatures, where acetic acid (CH_3_COOH) was used to dissolve struvite (instead of HNO_3_) to evaluate the effect of the dissolution media on the products formed. A single-phase semicrystalline Mg-WH was obtained at 200 °C, while a mixture of Mg-WH and HA was formed at 350 °C (Figure 1C). The crystalline phases identified were matched with HA (ICDD no. 00-009-0432) and Mg-WH (ICDD no. 01-070-2064).

The percentage of HA and Mg-WH in CaP(N-Am)200 were found to be 51% and 49%, respectively. Whereas, HA was found as the major phase (88%) and Mg-WH as the minor phase (12%) for the CaP(Ac-Am)350.

The crystallite sizes of HA and Mg-WH samples responsible for Bragg reflections in the (002) and (0210) planes, respectively, were calculated from XRD data using the Scherrer equation (Equation (3)) [61]. In all the experiments, the crystallite sizes of Mg-WH were found to be between 62 and 82 nm. The crystallite size of HA was found to be 16.8 nm for CaP(N-Am)200 where struvite was dissolved in HNO_3_ and the experiment was conducted at 200 °C. However, a significantly larger crystallite size for HA (i.e., 79.8 nm) formed for CaP(Ac-Am)350 when struvite was dissolved in CH_3_COOH and conducted at 350 °C.

The crystallinity index of HA and Mg-WH in the sample CaP(N-Am)200 were 0.1 and 13.8, respectively, whereas 13.4 and 13.8 were found for CaP(Ac-Am)350, respectively.

The percentage of phases, crystallite size, and crystallinity index of the samples produced are presented in Table 4.

#### 4.2.2. Morphology Characterisation Using FEG-SEM-EDX and TEM

**Figure 2:**
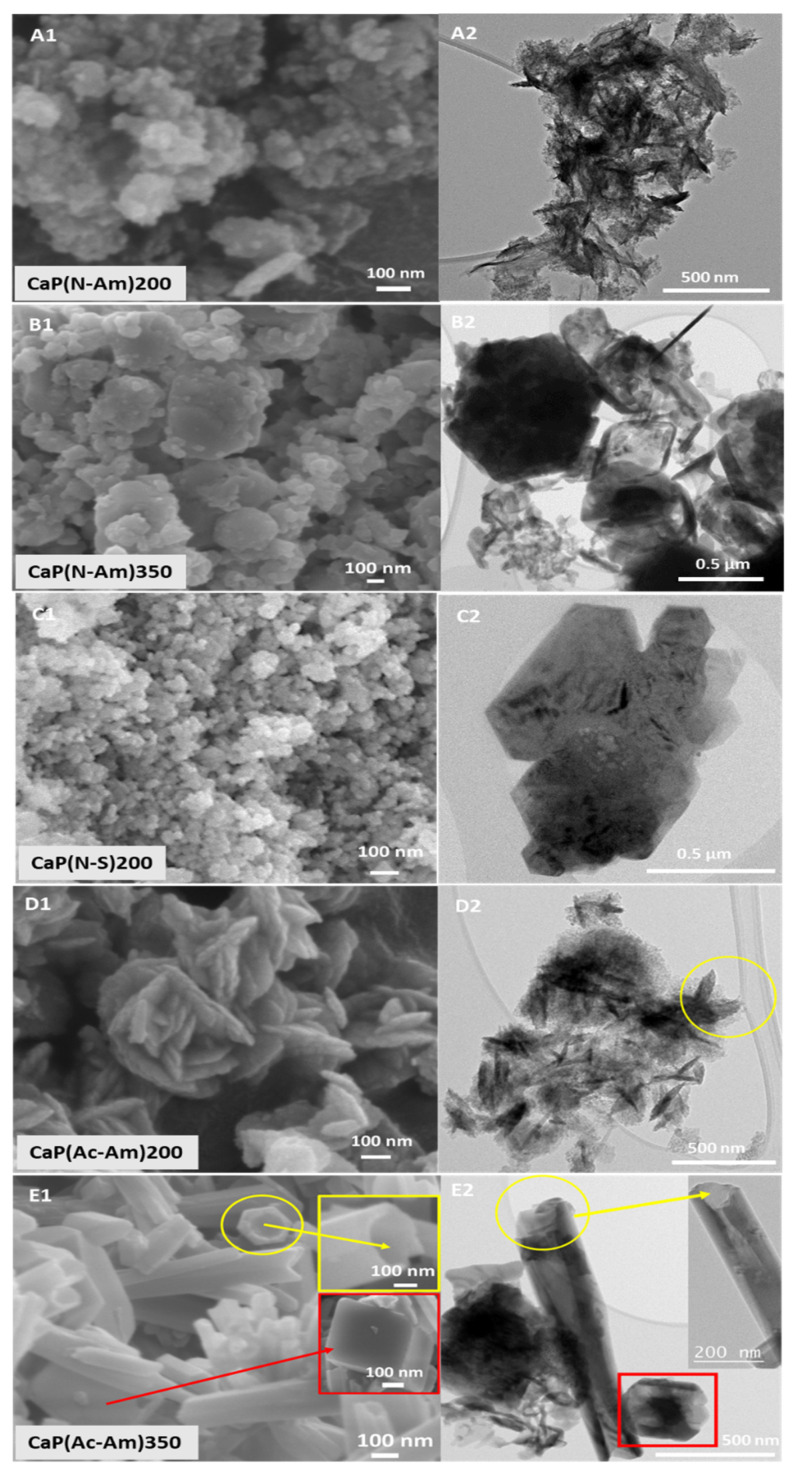
SEM and TEM images of samples synthesised using calcium nitrate tetrahydrate solution (pH = 11) and struvite solution (pH = 6) at 200 °C and 350 °C, where HNO_3_ (**A**–**C**) and CH_3_COOH (**D**,**E**) were used to dissolve struvite and NH_4_OH (**A**,**B**,**D**,**E**) and NaOH (**C**) were used to adjust the pH of the precursor solutions. (**E**) The yellow colour highlights the tube morphology observed and the red colour highlights rhombohedral-shaped particles.

Figure 2 represents the morphologies of the samples obtained as characterised by FEG-SEM (Figure 2A1–E1) and TEM (Figure 2A2–E2). When HNO_3_ was used to dissolve struvite and NH_4_OH was used to adjust the pH of the precursor solutions, nanoparticles formed at 200 °C, while larger particles (ranging from nano to submicron) were observed at a higher temperature of 350 °C. Using NaOH as a base solution, nano-sized homogeneous Mg-WH particles were produced. Whereas, using acetic acid and NH_4_OH as a base solution struvite solution was prepared; Mg-WH particles with plate shaped morphology were seen to have formed at 200 °C. Interestingly, the tubular morphology of HA along with rhombohedral-shaped Mg-WH particles were observed at a higher temperature (i.e., 350 °C) in this system. These morphologies can be clearly observed from the TEM images obtained (see Figure 2E2).

The particle sizes of the CaP(Ac-Am)350 were found to range between 50 and 160 nm in diameter and between a 20 and 40 nm tube thickness and between 23 and 720 nm for the rhombohedral-shaped particles. The particles in the remaining samples were found to be agglomerated as seen in Figure 2 and therefore, their particle sizes could not be accurately measured.

The Ca/P ratios for the samples CaP(N-Am)200 and CaP(Ac-Am)350 were obtained in the ranges between 1.42–1.65 and 1.45–1.77, while the (Ca + Mg)/P ratios were between 1.47–1.83 and 1.5–1.74, respectively, for these samples. The Ca/P and (Ca + Mg)/P ratios of rest of the samples were observed in the range of 1.23–1.3 and 1.4, respectively. The ratios of Ca/P and (Ca + Mg)/P of the samples prepared including their morphologies are presented in Table 5 and their elemental compositions are provided in Table S1 as supplementary materials.

#### 4.2.3. Functional Group Analysis by FTIR

**Figure 3:**
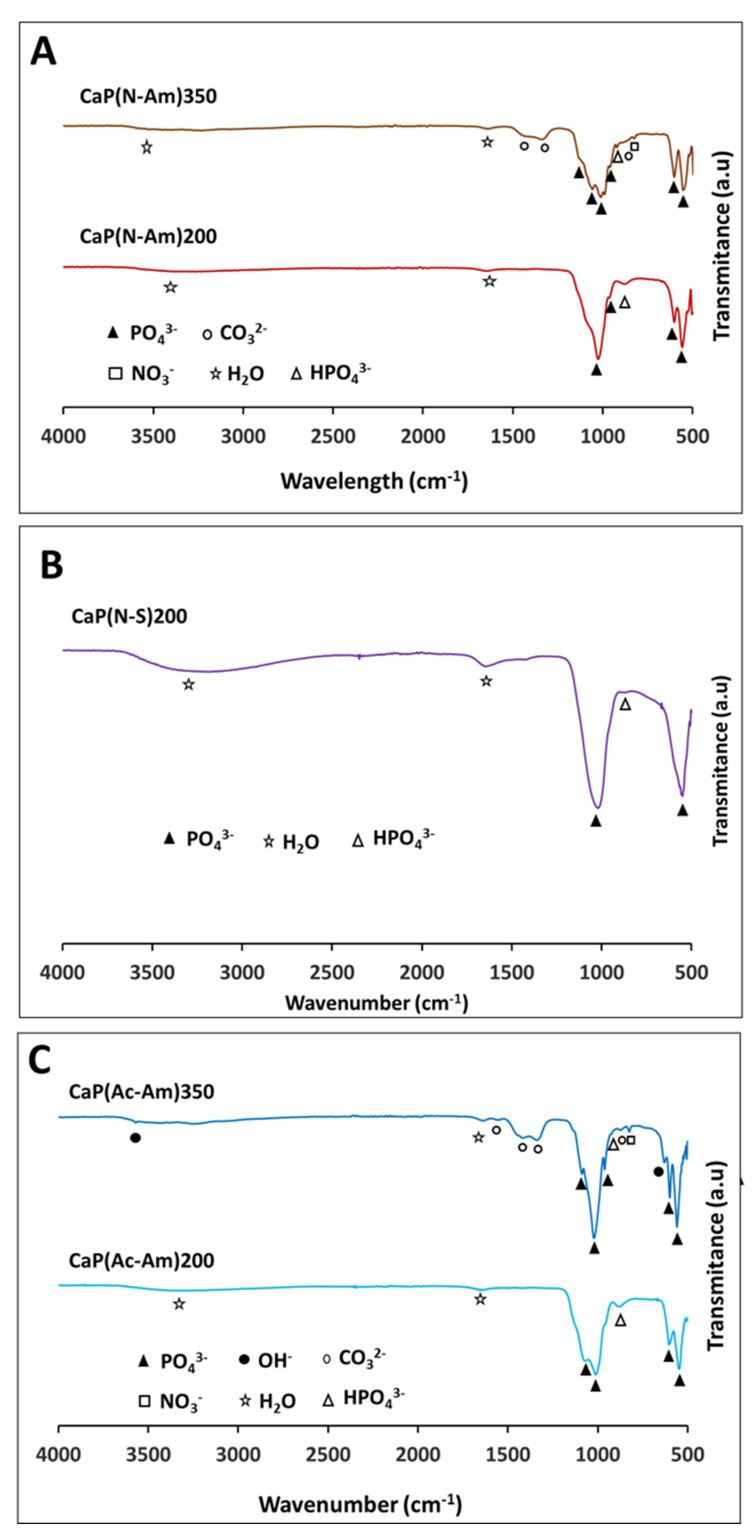
FTIR spectra of samples synthesised at 200 °C and 350 °C at constant pH of 11 and 6 for a calcium nitrate tetrahydrate solution and struvite solution, respectively, where acid and base solutions were as follows: (**A**) HNO_3_ and NH_4_OH, (**B**) HNO_3_ and NaOH, and (**C**) CH_3_COOH and NH_4_OH, respectively.

The FTIR spectra of samples prepared from the three sets of experiments are shown in Figure 3. Characteristic peaks related to different functional groups such as PO_4_^3−^, structural OH^-^, HPO_4_^2−^, and CO_3_^2−^ for all the samples prepared are presented in Table S2. The strong bands of PO_4_^3−^ modes were identified in the range of 960–1087 cm^−1^ and 547–603 cm^−1^ [87] for all the samples prepared. A noticeable structural OH^-^ band was observed at 629 cm^−1^ and 3570 cm^−1^ for the sample CaP(Ac-Am)350. The characteristic bands of HPO_4_^2−^ at around 870 cm^−1^ were observed for all the samples produced, while a band at around 920 cm^−1^ (related to HPO_4_^2−^) was identified only for the samples (CaP(N-Am)350 and CaP(Ac-Am)350) obtained at a higher temperature (350 °C). However, the band at around 875 cm^−1^ also corresponds to CO_3_^2−^ bending. The strong bands of CO_3_^2−^ in the range of 1338–1425 were observed for the samples prepared at high temperature, whereas a weak band at 1553 cm^−1^ was only appeared for CaP(Ac-Am)350. All the samples also showed a band related to absorbed water at around 1640 cm^−1^. The broad band in the range of 3000–3600 cm^−1^, corresponding to absorbed water, was also identified for all the samples except CaP(Ac-Am)350. In addition, a peak at ~823 cm^−1^, corresponding to NO_3_^−^ was found for the samples produced at a higher temperature (i.e., 350 °C).

### 4.3. Hydroxyapatite Synthesis Using Eggshell—Struvite

After having observed that mixed-phases of Mg-WH and HA or a single phase of Mg-WH could be obtained using calcium nitrate tetra-hydrate (as the Ca source) and struvite (as the P source), these were then replaced with potentially sustainable source for Ca (i.e., eggshells) and P (in struvite form) and introduced into a hydrothermal synthesis process.

#### 4.3.1. Investigating Phase Changes Using XRD

**Figure 4:**
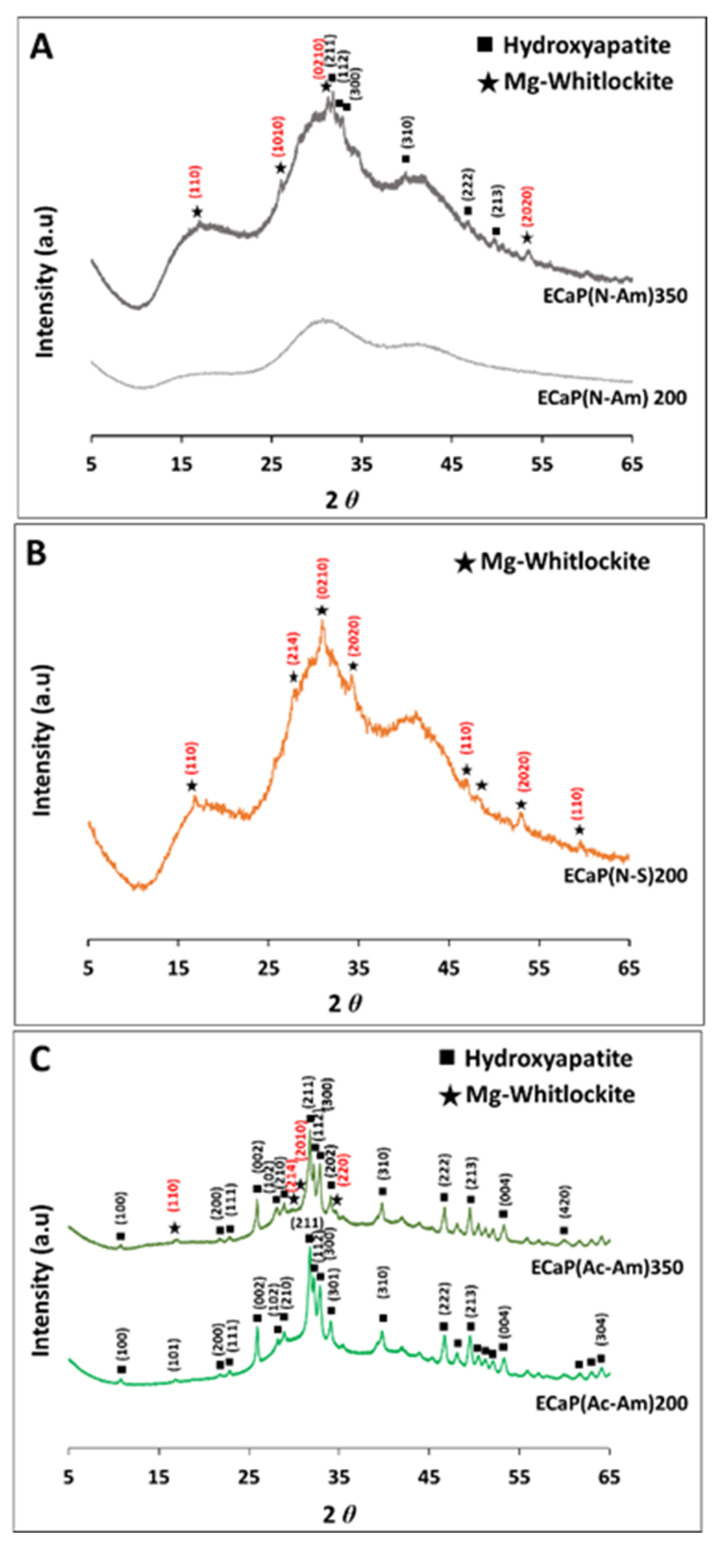
The XRD spectra of samples synthesised at 200 °C and 350 °C via a hydrothermal synthesis process at a pH of 11 of eggshell solution and a pH of 6 of struvite solution adjusted using (**A**,**C**) NH_4_OH (Am) and (**B**) NaOH (S) individually, where eggshells and struvite were dissolved in (**A**,**B**) HNO_3_ (N) and (C) CH_3_COOH (Ac).

The XRD spectra of the samples produced from the above two precursor solutions (i.e., eggshell and struvite solutions) at 200 °C and 350 °C are presented in Figure 4A–C. A well-pronounced background was observed for all the obtained XRD patterns. This observation revealed that the samples (i.e., ECaP(N-Am)350 and ECaP(N-S)200) mostly consisted of an amorphous phase along with a minor crystalline phase of biphasic CaP (HA and Mg-WH) for ECaP(N-Am)350 (Figure 4A) and a single phase of Mg-WH for ECaP(N-S)200 (Figure 4B). Whereas ECaP(N-Am)200 formed only amorphous calcium phosphate (ACP) at 200 °C (Figure 4A).

Figure 4C shows the XRD spectra of the samples produced at 200 and 350 °C, where acetic acid was used to dissolve eggshells and struvite (instead of HNO_3_) to evaluate the effect of acid on the samples formed. The pH of both solutions was adjusted using NH_4_OH_._

A single-phase of HA was obtained at 200 °C, whereas a multiphase of Mg-WH and HA formed at 350 °C (Figure 4C). The crystalline phases identified were matched with HA (ICDD No. 00-009-0432) and Mg-WH (ICDD No. 01-070-2064).

Samples containing multiphase were observed to be formed at the higher temperature of 350 °C, where 49% of Mg-WH and 51% of HA were found for the sample (ECaP(N-Am)350) produced using HNO_3_ to dissolve precursors. Whereas HA was seen to have formed as the major phase (81%) with a minor phase of Mg-WH (19%) when CH_3_COOH was used to dissolve the precursors (ECaP(Ac-Am)350) (Figure 4C).

Similar crystallite sizes of 80.6 and 81.7 nm were observed for HA and Mg-WH, respectively. Crystallinity index was 13.8 for all the samples. The percentage of phases, crystallite size and crystallinity index of the samples produced are presented in Table 6.

#### 4.3.2. Morphology Characterisation Using FEG-SEM-EDX and TEM

**Figure 5:**
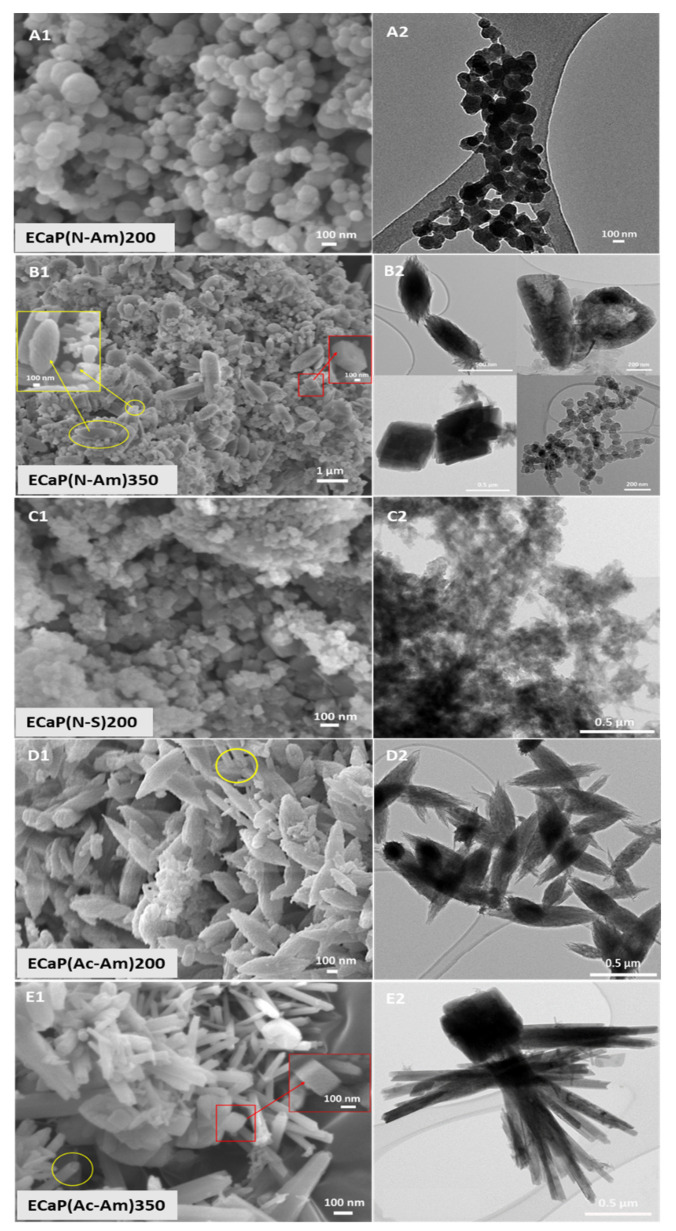
Surface morphology of samples synthesised using eggshell solution (pH = 11) and struvite solution (pH = 6) at 200 °C and 350 °C, where HNO_3_ (**A**–**C**) and CH_3_COOH (**D**–**E**) were used to dissolve eggshells and struvite, and NH_4_OH (**A**,**B**,**D**,**E**) and NaOH (**C**) were used to adjust the pH of the precursor solutions. The ellipsoidal and rod-shaped HA are highlighted in yellow colour and the rhombohedral-shaped Mg-WH is highlighted in red colour.

The surface morphology of the samples prepared was examined using FEG-SEM and TEM, as presented in Figure 5A1–E1 and Figure 5A2–E2, respectively. When HNO_3_ was used to dissolve precursors and NH_4_OH to adjust the pH of the precursor solutions, spherical-shaped particles were observed at 200 °C (Figure 5A), whereas only a few crystals of mixed morphologies consisting of ellipsoidal, tube, and rhombohedral shapes of HA and Mg-WH were found at a higher temperature (i.e., 350 °C) (Figure 5B). Irregular-shaped nanoparticles, including some rhombohedral crystals of Mg-WH, were observed at 200 °C when NaOH was used as a base solution (Figure 5C). Mixed morphologies of HA and/or Mg-WH were also observed for the samples obtained at both temperatures (i.e., 200 and 350 °C) using CH_3_COOH and NH_4_OH to dissolve precursors and adjust the pH, respectively. Larger particles (nano to submicron) of ellipsoidal and tubular (yellow circled) shapes of HA were observed at 200 °C (Figure 5D), whereas rod- and tube-shaped (yellow circled) HA and rhombohedral (red box and single crystal image in the inset) shapes of Mg-WH particles were found to form at 350 °C (Figure 5E).

From the TEM images, ellipsoidal, tube, and rhombohedral particles were clearly observed.

The particle size range of the samples measured from FEG-SEM images via ImageJ software is presented in Table 7. It was observed that most of the samples ranged from nano to submicron sizes. The particle sizes for ACP obtained at 200 °C were in the range of 28–196 nm. Ellipsoidal-shaped particles of ECaP(Ac-Am)200 were found to be between 96 and 273 nm. At a higher temperature (i.e., 350 °C), samples obtained using HNO_3_ were found to consist of ellipsoidal particles that produced size ranges of 273–522 nm width and 238–278 nm of rhombohedral and tubular particles with an inner diameter of 99–290 nm and a thickness of 28–71 nm. Whereas, using CH_3_COOH to dissolve precursors produced size ranges of 37–266 nm for tubular morphologies and 96–214 nm for rhombohedral morphologies. Particle sizes of ECaP(N-S)200 samples could not be measured due to agglomeration.

The Ca/P and (Ca + Mg)/P ratios for the samples ECaP(N-Am)350 and ECaP(Ac-Am)350 were found to be in the similar ranges, which were in between 1.23–1.5 and 1.6–1.7, respectively. The ratios of Ca/P and (Ca + Mg)/P of all the samples prepared including their morphologies and particle sizes are presented in Table 7 and their elemental compositions are provided in Table S3 as supplementary materials.

#### 4.3.3. Functional Group Analysis by FTIR

**Figure 6:**
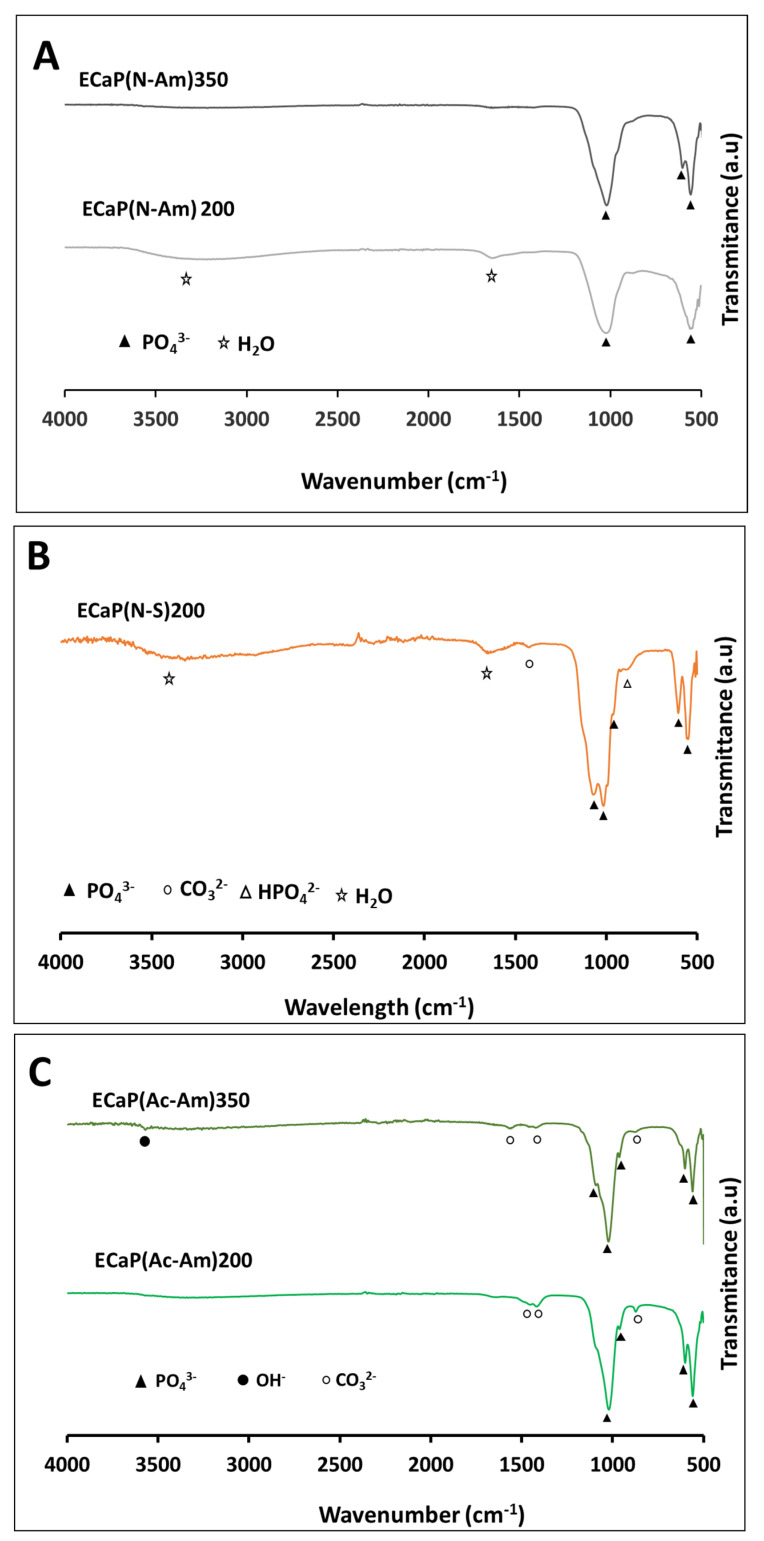
FTIR spectra of samples synthesised at 200 °C and 350 °C at constant pH of 11 and 6 for eggshell solution and struvite solution, respectively, where acid and base solutions were as follows: (**A**) HNO_3_ and NH_4_OH, (**B**) HNO_3_ and NaOH, and (**C**) CH_3_COOH and NH_4_OH, respectively.

Figure 6A–C represent the FTIR spectra of samples prepared from the three sets of experiments. The characteristics peak related to different functional groups such as PO_4_^3−^, structural OH^-^, HPO_4_^2−^, and CO_3_^2−^ for all the samples prepared are presented in Table S4.

All the samples showed characteristic bands of PO_4_^3−^ in the range of 550–602 cm^−1^ and 960–1092 cm^−1^ [87]. A significant band of structural OH^-^ was observed at 3572 cm^−1^ for the sample ECaP(Ac-Am)350. In addition, absorbed bands at around 875 cm^−1^, 1420–1450 cm^−1^ and 1562 cm^−1^ attributed to the CO_3_^2−^ were identified for the samples prepared using acetic acid, while a weak peak at 920 cm^−1^ corresponding to HPO_4_^2−^ was observed for ECaP(N-S)200. A broad band of absorbed water at 3000–3600 cm^−1^ along with a small band at 1651 cm^−1^ was identified for the sample ECaP(N-S)200 and ECaP(N-am)200.

## 5. Discussion

Waste materials from varying sectors and sources, such as farming or food processing, create adverse environmental effects such as soil, water, or air pollution [8]. However, the reuse of these waste materials would not only protect the environment but could also provide a valuable raw resource for the development of new materials. Eggshell-derived hydroxyapatite (HA) has been shown to have superior osteoconduction [88,89,90] and is more biocompatible with human bone due to the presence of some trace elements such as Na, Mg, P, Sr, and Zn [61,62]. Likewise, direct disposal of P-rich wastes and waste streams can cause eutrophication, subsequently resulting in contamination of fresh water streams [73]. Thus, phosphorus recovery in the form of struvite is a sustainable approach towards resource scarcity as well as environmental protection. Kataki et al. [67] reported that commercially available struvite recovery technologies were becoming available (i.e., Pearl^®^ and/or ASSTRIP^®^ (USA), Phospaq^TM^ (The Netherlands), Phosnix (Japan), Seaborne (Germany), AirPrex^TM^ (Germany, Netherlands), and Multiform (America)) reporting up to 80–90% P recovery.

To explore the potential use of struvite as a P precursor, initial trials were conducted using a conventional Ca source (CaNO_3_.4H_2_O) first, then followed by a replacement with eggshells as a sustainable source to prepare CaPs via a hydrothermal synthesis process.

### 5.1. Samples Prepared from Calcium Nitrate Tetrahydrate and Struvite Solutions

Pure magnesium whitlockite (Mg-WH) and/or biphasic Mg-WH and HA were observed to have formed (as confirmed via XRD, EDX, and FTIR analysis) using calcium nitrate tetrahydrate and struvite solutions as precursors. The pH of 6 and pH of 11 were maintained using NH_4_OH and NaOH (1M) for struvite solutions and CaNO_3_.4H_2_O solutions, respectively. A higher pH of struvite solution could not be prepared due to precipitation occurring at around pH 7. The solubility of struvite depends on temperature and pH [91]. Ariyanto et al. [91] reported that the solubility of struvite decreased with increasing pH until it reached a minimum solubility at pH 7.4. However, the solubility of struvite increases above a pH of 9. Moreover, they also observed that its solubility increased from 25 °C to 35 °C and decreased at 40 °C [91]. The dissolution of struvite between 25°C and 35 °C required energy (endothermic) for the solute particle to enter the solution. Increasingly, solute particles in solution increased the solubility of the solid. Above 35 °C, the dissolution of a solid releases heat, and its solubility decreases as the temperature increases [91].

The formation of Mg-WH, in all cases, was due to the presence of magnesium in the struvite. Studies report that Mg-WH formed when Mg^2+^ ions are present in acidic solutions containing calcium phosphate. In addition, in vivo formation of Mg-WH occurred under acidic conditions via the release of acidic molecules when osteoclasts resorbed old bone [33]. Jang et al. [92] also confirmed that HA can directly transform into Mg-WH under acidic pH conditions with sufficient amounts of Mg^2+^ ions. In this study, the pH of the CaP suspensions formed was found to be in the range of 5–7, which was found to be the ideal condition for the formation of Mg-WH and/or a mixture of Mg-WH and HA.

The formation of CaP phases at different temperatures (200 and 350 °C) was strongly dependent on the acids used to prepare the struvite solution (see Figure 1A,C). When NH_4_OH was utilized as a pH buffer and HNO_3_ was used to dissolve struvite, almost every part of Mg-WH (49%) and HA (51%) were formed at a lower temperature of 200 °C, whereas a single phase of Mg-WH formed at a higher temperature (Figure 1A). Studies show that magnesium can inhibit the formation of apatite [93]. Substitution of magnesium for calcium in the apatite structure inhibits crystallisation by structural deformation of the crystal nuclei [94]. A higher crystalline phase of pure Mg-WH and/or biphasic CaP (Mg-WH and HA) were observed at a higher temperature (Figure 1A,C) due to the increase of crystal size with increasing temperature [95].

However, an opposite phenomenon was observed in this study when acetic acid was used instead of HNO_3_ to dissolve the struvite. Poorly crystalline Mg-WH formed at 200 °C and HA (88%) with a secondary phase of Mg-WH (12%) formed at 350 °C (Figure 1C). This phenomenon was due to the higher dissociation capability of a strong acid (HNO_3_), which could dissolve struvite completely and provide more Mg^+2^ in the precursor solution compared to weak acid (CH_3_COOH).

The formation of nanocrystalline HA (16.8 and 79.8 nm) and Mg-WH (62–81.6 nm) were initially assessed based on the calculation of crystallite at size (002) and (0210) plane for HA and Mg-WH, respectively, from the XRD analysis (see Table 4). The literature shows that the structural analysis of synthesised HA is usually focused on the (002) reflection at 25.8 (2θ) and the planes (112 and 300) between 30° and 35° (2θ) [18,19]. However, poor resolution of peaks in the later range (30°–35° (2θ)) is usually observed for low-crystalline materials, and hence it can be difficult to distinguish between them [18,19]. A similar phenomenon was found for the sample CaP(N-Am)200 in this study, as shown in Figure 1A, where low resolution of the peaks between 30° and 35° (2θ) (at the (112) and (300) plane) were observed and could hardly be differentiated. On the contrary, because of the high resolution and intensity of the (002) reflection, the FWHM of the (002) reflection could be measured more precisely, and therefore the crystallite size and crystallinity index were measured at the (002) reflection for the HA of all samples in this study. The crystallinity index of the samples was observed to increase with increasing crystallite size. In a study of the crystallinity index of HA, Sa et al. [18] reported that there was a strong linear correlation between crystallinity index and crystal size. This correlation was due to the HA containing a large crystallite size and presenting a narrow peak in the XRD diffractogram, resulting in a small FWHM as well as a large crystallinity index.

In our study, the Ca/P ratios for CaP(N-Am)350, CaP(N-S)200, and CaP(Ac-Am)200 were found to be in the range between 1.23 and 1.3, while the (Ca + Mg)/P element molar ratio of all these samples produced was 1.4, which was close to the standard Mg-WH powder (Ca_18_Mg_2_(HPO_4_)_2_(PO_4_)_12_) with (Ca + Mg)/P of 1.43 [96]. This finding confirms the presence of Mg-WH in these samples. However, a slight increase of the (Ca + Mg)/P ratios was observed in the range of 1.47–1.83 for CaP(N-Am)200, suggesting formation of HA along with Mg-WH due to more Ca^2+^ present in the product suspension. Similarly, the higher (Ca + Mg)/P ratio of CaP(Ac-Am)350 confirm the higher percentage of the HA phase formation.

When HNO_3_ media was used for dissolution, irregularly shaped particles, including plates (approximately > 30 nm) formed at lower temperature of 200 °C as as confirmed via FEG-SEM and TEM analyses (Figure 2A). While larger particles of rhombohedral shape formed (Figure 2B) at a higher temperature (350 °C). A similar rhombohedral morphology of the Mg-WH was also observed by Jang et al. where whitlockite was synthesised via a precipitation method from calcium hydroxide (Ca(OH)_2_), magnesium hydroxide (Mg(OH)_2_), and phosphoric acid (H_3_PO_4_) in an aqueous system at 80 °C [97]. Using acetic acid to dissolve struvite, plate-shaped Mg-WH formed at a lower (200 °C) temperature (Figure 2D), and interestingly, the tubular morphology of HA (50–160 nm diameter and 20–40 nm thickness) along with rhombohedral Mg-WH (23–720 nm) formed at a higher temperature of 350 °C (Figure 2E2). As such, the crystallinity, particle size, and morphology of the CaPs synthesised were found to be dependent on the reaction temperature. However, these morphologies also seemed to depend on the acids used to dissolve the struvite (precursor) and the pH buffers used.

The formation of HA and Mg-WH also correlated with the FTIR analyses.

Literature reported that the presence of PO_4_^3−^ in the ranges between 530–610 and 900–1100 cm^−1^ and OH^−^ group at around 630 cm^−1^ and 3568 cm^−1^ confirmed HA formation [93]. The sharp and intense peaks of PO_4_^3−^ and OH^−^ within these ranges appeared for CaP(Ac-Am)350 (where acetic acid was used to dissolve struvite), which indicated the formation of HA as the main phase (also confirmed via XRD). 

A reduction in the resolution of these bands for the remaining samples indicated the formation of Mg-WH as a prominent phase with the decrease in the HA content. These results correlated with the FTIR spectrum of biological HA, as reported by Lin et al., who stated that the gradual merging of PO_4_^3−^ peaks and disappearance of OH^-^ mode and stretching bands at around 634 and 3571 cm^−1^ occurred with the formation of Mg-WH [96].

The presence of characteristic bands at 1552–1554 cm^−1^ associated with A-type CO_3_^2−^and the absorption bands at around 874 cm^−1^ and 1417 cm^−1^ assigned to B-type CO_3_^2−^vibration mode v2 and v3 for sample CaP(Ac-Am)350 suggested that a certain amount of CO_3_^2−^ ions were present in the both the OH^-^ site and PO_4_^3–^tetrahedra within the HA structure. Similar findings for the presence of CO_3_^2−^ ions in the PO_4_^3–^ tetrahedra within the HA structure were reported by Lin et al. [96]. They observed that bands at 1562 and 1582 cm^−1^ were associated with A-type CO_3_^2−^ and B-type CO_3_^2−^at 878 cm^−1^ and in the region of 1411–1450 cm^−1^ [96]. The existence of the CO_3_^2−^ could have been due to impurities in the starting materials, probably due to not being sufficiently degassed prior to use. This suggested that nonstoichiometric CO_3_^2−^ containing hydroxyapatite had formed. Moreover, appearance of low intensity bands HPO_4_^2−^ at around 874 and/or 922 cm^−1^ were in good agreement with increasing Mg-WH formation and content [98].

Bands assigned for NO_3_^−^ deformation (*v_2_*) at 825 (826 and 831) cm^−1^ were only present in the case of CaP(N-Am)350 and CaP(Ac-Am)350, which suggested that some residue from the precursors or reactants remained. Kannan and co-workers reported that the band at 875 cm^−1^ could be assigned to the presence of nitrates and other residual species, such as NO_3_^−^ and NH_4_^+^ possibly originating from precursors or reactants used during the synthesis [93]

### 5.2. Samples Prepared from Eggshell Solution and Struvite Solution

The diffraction pattern of the samples prepared using HNO_3_ to dissolve both precursors and the NH_4_OH solution to maintain the pH showed the formation of an amorphous calcium phosphate phase (Figure 4A) containing Mg (Table 7) at 200 °C, whereas the poorly crystalline peaks of Mg-WH along with HA as a secondary phase were observed at a higher temperature of 350 °C (Figure 4A). This occurrence may be due to the transformation of ACP to Mg-WH and HA at a higher temperature [99]. The XRD results were correlated to the FEG-SEM images (Figure 5), where the formation of crystal structures of ellipsoidal and tubular HA along with rhombohedral Mg-WH within the amorphous phase were observed at a higher temperature, as confirmed via TEM analysis.

One the other hand, poorly crystalline homogenous Mg-WH formed at 200 °C, when NaOH was used as the pH buffer instead of the NH_4_OH solution. Some irregularly shaped crystals within the amorphous structure were also observed via FEG-SEM and TEM (Figure 4B). Therefore, NH_4_OH also showed a significant effect here on the formation of different morphologies compared to NaOH.

Interestingly, phase-pure, homogenous, and ellipsoidal-shaped Mg-containing HA was seen to have formed at a lower temperature (200 °C), whereas a secondary phase of HA along with Mg-WH formed at a higher temperature (350 °C), when precursor solutions were dissolved using acetic acid instead of nitric acid and NH_4_OH was the pH buffer. This finding could be attributed to the different ionic concentrations (especially Ca^2+^ and Mg^2+^) in solution when struvite was dissolved in strong HNO_3_ and weak acetic acid and the variation of operating temperatures (200 and 350 °C). In homogeneous precipitation conditions, unstable ACP formed first, and two possible transformation approaches could have potentially occurred according to the pH conditions [100]. When the pH ranges from 7 to 9, ACP is initially transformed into a precursor octacalcium phosphate phase and then into the HA phase [100]. In contrast, when the pH is higher than 9, the intermediate conversion step is bypassed and amorphous calcium phosphate converts directly to HA [100]. As such, HA and/or amorphous calcium phosphates could have co-precipitated with Mg-WH from the solutions simultaneously in the presence of calcium and Mg ions. Moreover, the pH was confirmed to have an important role for the precipitation of both Mg and calcium phosphate as both the HA and Mg-WH formation were found to be pH sensitive [92,94]. Hydroxyapatite is known as the most thermodynamically stable CaP phase at near-neutral pH values [94], and Mg-WH is one of the most thermodynamically stable Mg-incorporated calcium phosphate compounds in acidic conditions [92]. In this study (2^nd^ experimental section), the final product pH for all the samples produced using NH_4_OH was about 9, and pH ~ 5 was observed when NaOH was used as a pH buffer. These values justified the formation of HA as the predominant phase and phase pure Mg-WH in the case of using NH_4_OH and NaOH, respectively, using a hydrothermal synthesis process.

The EDX data of all the samples were also in good agreement with the XRD results. The ranges of Ca/P and (Ca + Mg)/P ratios (1.23–1.5 and 1.6–1.7, respectively) for ECaP(Ac-Am)350 and ECaP(N-Am)350 confirmed the presence of both the HA and Mg-WH phases. The Ca/P and (Ca + Mg)/P ratios (1.3 and 1.4, respectively) for ECaP(N-S)200 indicated the formation of a single-phase of Mg-WH, while these ratios of 1.6 and 1.7, respectively, suggested the presence of Mg containing HA in ECaP(Ac-Am)200. In addition, the Ca/P and (Ca + Mg)/P ratios (0.78 and 1.45, respectively) for ECaP(N-Am)200 indicated the formation of Mg containing ACP [101].

These XRD results also correlated with the FTIR analyses (Figure 6), where higher resolution peaks of PO_4_^3−^ were observed when acetic acid was used to dissolve the precursors, resulting in the higher crystalline phase formation of HA. Moreover, the observation of the characteristic OH^-^ peak at 3572 cm^−1^ only for ECaP(Ac-Am)350 indicated the formation of highly crystalline HA. In the case of using nitric acid, absorption bands of PO_4_^3−^ were observed to have merged into a broad peak at around 500–600 cm^−1^ and 900–1100 cm^−1^. The reduction of PO_4_^3−^resolution with the disappearance of OH^-^ peak, indicated the decrease in the HA content with the presence of Mg-WH in the sample ECaP(N-Am)350 and the amorphous phase of ACP in the sample. On the other hand, the observation of HPO_4_^2-^ group at 920 cm^−1^ only for ECaP(N-S)200 suggested the formation of only Mg-WH. The presence of CO_3_^2-^ in the region 1420–1450 cm^−1^ with a weak peak at around 875 cm^−1^ for the samples ECaP(Ac-Am)200 and ECaP(Ac-Am)350 confirmed carbonated apatite formation with a B-type substitution in the apatite structure; however, a weak band at 1562 cm^−1^ was observed for the later sample which indicated that both A and B-type substitution had taken place [36,93].

The crystallite sizes of all the samples were found to be similar (80.6–81.7 nm), which revealed the nanocrystalline structure of the developed HA and Mg-WH. A similar value of crystallinity index (13.8) was observed for all the developed samples; this similarity was due to the lack of significant variation in crystallite sizes, as discussed above.

The outcomes from this study revealed that useful products of phase-pure HA and Mg-WH or mixtures of HA and Mg-WH could be prepared from waste bioresources using eggshells and struvite by altering the parameters of a hydrothermal synthesis process. These materials could potentially be used for bone repair and regeneration, as Mg-WH is also present in natural bone. In a recent study, Mg-WH showed better osteogenic activity and increased bone formation when compared to HA due to the continuous release of magnesium and phosphate ions [102]. Moreover, the presence of both HA and Mg-WH at an approximate 1:3 ratio was shown to have promoted osteogenic activity [33]. However, further in vitro and pre-clinical in vivo studies would be required to confirm the efficacy of these materials for bone repair indications.

## 6. Conclusions

Waste-derived materials such as eggshells and struvite (as sources of calcium and phosphorus, respectively) along with acetic acid as the dissolution media were utilised in this study and shown to produce calcium phosphates (CaPs) with the goal of sustainable manufacturing of biomaterials. Nanocrystalline pure magnesium whitlockite (Mg-WH) or hydroxyapatite (HA) and/or biphasic Mg-WH and HA formed via a hydrothermal synthesis process as confirmed via XRD, FEG-SEM, TEM, EDX, and FTIR analysis were studied. Different morphologies of the nanocrystalline HA and Mg-WH could be achieved by controlling the process parameters and the selection of dissolution media and base solutions for adjusting the pH. For example, the tubular morphology of HA (37–266 nm diameter) along with rhombohedral Mg-WH (96–214 nm) were formed at a higher temperature (350 °C), while the ellipsoidal-shaped particles (96–273 nm) of HA were formed at 200 °C using acetic acid as a dissolution media. The outcomes of this study indicated that struvite could be a potentially useful P source to prepare CaP-based biomaterials with minimal usage of synthetic chemicals. However, bioactivity characterisation and pre-clinical in vivo studies would be required to confirm the efficacy of these biowaste-derived biomaterials.

## Figures and Tables

**Table 1:** The five samples synthesised via a hydrothermal synthesis process at 200 and 350 °C, with pH 11 of calcium nitrate tetrahydrate [Ca (NO_3_)_2_.4H_2_O] solution and pH 6 of struvite solution, along with their respective codes, are highlighted below. The pH of both solutions was adjusted using NH_4_OH and NaOH. CaP = calcium phosphate, N = HNO_3_, Am = NH_4_OH, and S = NaOH.

Sample Names	Acid to Dissolve Struvite	pH Buffer	Temperature
CaP(N-Am)200	HNO_3_ (N)	NH_4_OH (Am)	200 °C
CaP(N-Am)350			350 °C
CaP(N-S)200		NaOH (S)	200 °C
CaP(Ac-Am)200	CH_3_COOH (Ac)	NH_4_OH (Am)	200 °C
CaP(Ac-Am)350			350 °C

**Table 2:** Samples with respective codes synthesised via a hydrothermal synthesis process at 200 and 350 °C at pH 11 of eggshell solution and at a constant pH 6 of struvite solution are shown. The pH of both solutions was adjusted using NH_4_OH and NaOH. ECaP = eggshell-derived calcium phosphate, N = HNO_3_, Ac = CH_3_COOH, Am = NH_4_OH, and S = NaOH.

Sample Names	Acid to Dissolve Struvite	pH Buffer	Temperature
ECaP(N-Am)200	HNO_3_ (N)	NH_4_OH (Am)	200 °C
ECaP(N-Am)350			350 °C
ECaP(N-S)200		NaOH (S)	200 °C
ECaP(Ac-Am)200	CH_3_COOH (Ac)	NH_4_OH (Am)	200 °C
ECaP(Ac-Am)350			350 °C

**Table 3:** Compositions of pre-treated eggshells via ICP-MS.

Components	Mol%	Wt%
Ca	97.11 ± 0.23	98.13 ± 0.14
Na	0.56 ± 0.08	0.32 ± 0.04
Mg	1.89 ± 0.08	1.16 ± 0.05
P	0.40 ± 0.08	0.31 ± 0.06
Sr	0.02 ± 0.01	0.04 ± 0.01
Zn	0.02 ± 0.01	0.03 ± 0.01

**Table 4:** Crystallite size and crystallinity index at (002) and (0210) plane for HA and Mg-WH, respectively, including percentage of phases of the products (for the mixed phases) of samples prepared from calcium nitrate tetrahydrate solution and struvite solution via a hydrothermal synthesis process at 200 and 350 °C.

Sample Names	Products (Percentage of Phases)	Crystallite Size (nm)		Crystallinity Index	
		at the plane (002) for HA	at the plane (0210) for Mg-WH	at the plane (002) for HA	at the plane (0210) for Mg-WH
CaP(N-Am)200	HA (51%) + Mg-WH (49%)	16.8	81.6	0.1	13.8
CaP(N-Am)350	Mg-WH	-	81.6	-	13.8
CaP(N-S)200	Mg-WH	-	81.6	-	13.8
CaP(Ac-Am)200	Mg-WH	-	62	-	5.8
CaP(Ac-Am)350	HA (88%) + Mg-WH (12%)	79.8	81.6	13.4	13.8

**Table 5:** The morphology including the particle sizes and the ratios of Ca/P and (Ca + Mg)/P of the samples prepared from calcium nitrate tetrahydrate solution and struvite solution via a hydrothermal synthesis process at 200 and 350 °C.

Samples	Morphology (Particle Size)	Ca/P	(Ca + Mg)/P
CaP(N-Am)200	Irregular along with plate shape	1.42–1.65 (Avg. 1.51 ± 0.25)	1.47–1.83 (Avg. 1.66 ± 0.19)
CaP(N-Am)350	Rhombohedral	1.3 ± 0.05	1.4 ± 0.04
CaP(N-S)200	Rhombohedral	1.23 ± 0.01	1.39 ± 0.00
CaP(N-Am)200	Platelet	1.3 ± 0.01	1.4 ± 0.03
CaP(Ac-Am)350	Hexagonal tube (50–160 nm diameter with 20–40 nm thickness) + Rhombohedral (23–720 nm)	1.45–1.77 (Avg. 1.67 ± 0.16)	1.5–1.74 (Avg. 1.72 ± 0.13)

**Table 6:** Crystallite size and crystallinity index at (002) plane and (0210) for HA and Mg-WH, respectively, including percentage of phases of the products (for the mixed phases) of samples prepared from eggshell solution and struvite solution via a hydrothermal synthesis process at 200 and 350 °C.

Sample Names	Product (Percentage of Phases)	Crystallite Size (nm)		Crystallinity Index	
		at the plane (002) for HA	at the plane (0210) for Mg-WH	at the plane (002) for HA	at the plane (0210) for Mg-WH
ECaP(N-Am)200	-	-	-	-	-
ECaP(N-Am)350	HA (51%) + Mg-WH (49%)	-	81.6	-	13.8
ECaP(N-S)200	Mg-WH	80.6	-	13.8	-
ECaP(Ac-Am)200	HA	80.6	81.7	13.8	13.8
ECaP(Ac-Am)350	HA (81%) + Mg-WH (19%)	80.6	81.6	13.8	13.8

**Table 7:** The morphology including the particle sizes and the ratios of Ca/P and (Ca + Mg)/P of the samples prepared from eggshell solution and struvite solution via a hydrothermal synthesis process at 200 and 350 °C.

Samples	Crystal Morphology (Particle Size)	Ca/P	(Ca + Mg)/P
ECaP(N-Am)200	Spherical (28–196 nm)	0.78 ± 0.04	1.45 ± 0.05
ECaP(N-Am)350	Ellipsoidal (width 273–522 nm) Tube (inner diameter 99–290 thickness 28–71 nm) Rhombohedral (238–278 nm)	1.23–1.5 (Avg. 1.24 ± 0.12)	1.6–1.7 (Avg. 1.6 ± 0.08)
ECaP(N-S)200	Irregular-shaped along with rhombohedral	1.3 ± 0.03	1.4 ± 0.00
ECaP(Ac-Am)200	Ellipsoidal (96–273 nm)	1.6 ± 0.03	1.7 ± 0.03
ECaP(Ac-Am)350	Rod, Tube (37–266 nm diameter) Rhombohedral (96–214 nm)	1.3–1.5 (Avg. 1.46 ± 0.04) 1.48	1.6–1.7 (Avg. 1.68 ± 0.06)

## Data Availability

The original contributions presented in the study are included in the article/Supplementary Material, further inquiries can be directed to the corresponding author.

## Supplementary Materials

The following supporting information can be downloaded at: https://www.mdpi.com/article/10.3390/ma16062138/s1. Table S1: Elemental composition of samples produced at 200 °C and 350 °C from calcium nitrate tetrahydrate and struvite solutions. Table S2: FT-IR band positions and their corresponding assignments of samples produced at 200 °C and 350 °C from calcium nitrate tetrahydrate and struvite solutions [103,104,105,106,107,108,109,110,111,112,113,114]. Table S3: Elemental composition of samples produced at 200 °C and 350 °C from eggshell and struvite solutions. Table S4: FT-IR band positions and their corresponding assignments of samples produced at 200 °C and 350 °C from eggshell and struvite solutions [103,104,105,106,107,108,109,110,111,112,113].
