# Supplementary material for: Production of Nano Hydroxyapatite and Mg-Whitlockite from Biowaste-Derived products via Continuous Flow Hydrothermal Synthesis: A Step towards Circular Economy
Source: Materials (Basel). 2023 Mar 7;16(6):2138. doi: 10.3390/ma16062138 (PMC10058175; doi:10.3390/ma16062138)
Supplement: Supplementary file 1 [file materials-16-02138-s001.zip › materials-2221736-supplementary.pdf]

materials

MDPI

Supplementary Materials

Production of Nano Hydroxyapatite and Mg-Whitlockite from Biowaste-Derived products via Continuous Flow Hydrothermal Synthesis: A Step Towards Circular Economy

Farah Nigar[a,b], Amy-Louise Johnston[a,c], Jacob Smith[a,c], William Oakley[a], Md Towhidul Islam [d,e], Reda Felfel[f,g], David Grant[a], Edward Lester [a] and Ifty Ahmed [a*]

[a]*Advanced Materials Research Group, Faculty of Engineering, University of Nottingham, Nottingham, NG7 2RD, UK*
[b]*Bangladesh Council of Scientific and Industrial Research (BCSIR), Dhaka – 1205, Bangladesh*
[c]*Food Water Waste Research Group, Faculty of Engineering, University of Nottingham, Nottingham NG7 2RD, UK*
[d] *School of Physical Sciences, University of Kent, Canterbury, CT2 7NZ, UK*
[e] *Department of Applied Chemistry and Chemical Engineering, Faculty of Engineering, Noakhali Science and Technology University, Noakhali-3814, Bangladesh*
[f]*Department of Mechanical and Aerospace Engineering, Faculty of Engineering University of Strathclyde, Glasgow, G1 1XJ*
[g] *Physics Department, Faculty of Science, Mansoura University, Mansoura 35516, Egypt*
**Corresponding author:* ifty.ahmed@nottingham.ac.uk

Table S1: Elemental composition of samples produced at 200 ^oC and 350 ^oC from calcium nitrate tetrahydrate and struvite solutions.

Samples name	Elemental composition (atomic %)			
	O	Ca	P	Mg
**CaP(N-N)200**	66.33 ± 8.40	19.14 ± 6.72	12.68 ± 2.32	1.85 ± 0.59
**CaP(N-N)350**	66.95 ± 2.16	17.90 ± 1.51	13.77 ± 0.76	1.38 ± 0.04
**CaP(N-S)200**	65.97 ± 0.10	17.48 ± 0.30	14.1 ± 0.06	2.45 ± 0.20
**CaP(Ac-N)200**	69.21 ± 0.0	16.68 ± 0.10	12.83 ± 0.10	1.28 ± 0.20
**CaP(Ac-N)350**	65.54 ± 2.05	21.14 ± 3.16	12.66 ± 1.75	0.66 ± 1.53

Table S2: FT-IR band positions and their corresponding assignments of samples produced at 200 ^oC and 350 ^oC from calcium nitrate tetrahydrate and struvite solutions.

Observed band positions (cm^{-1})					Corresponding assignments	References
**CaP(N-Am)200**	**CaP(N-Am)350**	**CaP(N-S)200**	**CaP(Ac-Am)200**	**CaP(Ac-Am)350**		
557, 602	553, 603	551	547, 603	559, 600	PO_4^{3-} bending (v_4)	[93,103–106]
-	-	-	-	629	structural OH^-	[106,107]
-	825	-	-	825	NO_3^- out-of-plane deformation (v_2)	[106,108]
877	889	870	881	874	HPO_4^{2-} group, / B-site CO_3^{2-} bending	[87,105,106,108–113]
-	922	-	-	925	HPO_4^{2-} group	[15]
962	960	-	-	962	PO_4^{3-} bending (v_1)	[105,106,109,111]
1024	991, 1011, 1059	1022	1011, 1070	1020, 1087	PO_4^{3-} bending (v_3)	[105,109,111]
-	1340	-	-	1338	CO_3^{2-} bending (v_3)	[114]
-	1425	-	-	1417	B-site CO_3^{2-} bending (v_3)	[87,108–112]
-	-	-	-	1552 - 1554	A-type CO_3^{2-} bending	[93,106]
1638, 3100 - 3500	1641, 3100 - 3500	1649, 3000 – 3600	1641, 3100 – 3500	1641	Absorbed water	[93,106,109]
-	-		-	3570	Structural OH^-	[106]

Table S3: Elemental composition of samples produced at 200 ^oC and 350 ^oC from eggshell and struvite solutions.

Samples name	Elemental composition (atomic %)			
	**O**	**Ca**	**P**	**Mg**
**ECaP(N-N)200**	71.69 ± 2.28	8.87 ± 0.45	11.37 ± 1.19	8.07 ± 0.71
**ECaP(N-N)350**	75.89 ± 4.86	11.54 ± 3.24	9.31 ± 1.68	3.26 ± 0.41
**ECaP(N-S)200**	64.64 ± 4.03	18.48 ± 2.47	14.55 ± 1.63	2.33 ± 0.14
**ECaP(Ac-N)200**	64.85 ± 0.56	20.79 ± 0.79	13.24 ± 0.46	1.12 ± 0.37

**ECaP(Ac-N)350**	71.48 ± 8.51	15.58 ± 6.8	10.66 ± 4.47	2.28 ± 1.34

Table S4: FT-IR band positions and their corresponding assignments of samples produced at 200 °C and 350 °C from eggshell and struvite solutions.

**Observed band positions (cm^{-1})**					**Corresponding assignments**	**References**
**ECaP(N-Am)200**	**ECaP(N-Am)350**	**ECaP(N-S)200**	**ECaP(Ac-Am)200**	**ECaP(Ac-Am)350**		
561	557, 601	550, 604	561, 601	561, 602	PO_4^{3-} bending (v_4)	[93,103–106]
-	-	-	874	877	HPO_4^{2-} group/ CO_3^{2-} bending (v_3)	[87,105,106,108–113]
-	-	920	-	-	HPO_4^{2-} group	[113]
-	-	962	962	960	PO_4^{3-} bending (v_1)	[93,105,106,109,111]
1022	1020	1014, 1070	1022	1022, 1092	PO_4^{3-} bending (v_3)	[105,109,111]
-	-	1423	1420, 1454	1423, 1452	B-type CO_3^{2-} bending (v_3)	[108–112]
-	-	-	-	1562	A-Type CO_3^{2-} bending (v_3)	[105,106]
1651, 3000 – 3600	-	1649, 3000 - 3600	-	-	Absorbed water	[105,106,109]
-	-	-	-	3572	Structural OH^-	[106]

References

87. Xu, G.; Aksay, I.A.; Groves, J.T. Continuous crystalline carbonate apatite thin films. A biomimetic approach. *J. Am. Chem. Soc.* **2001**, *123*, 2196–2203.
93. Li, J. Structural Characterisation of Apatite-Like Materials. Ph.D. Thesis, University of Birmingham, Birmingham, UK, 2010.
103. LeGeros, R.Z. Calcium phosphates in oral biology and medicine. Monographs in oral sciences **1991**, *15*, 109–111.
104. Tas, A.C. Synthesis of biomimetic Ca-hydroxyapatite powders at 37 C in synthetic body fluids. Biomaterials **2000**, *21*, 1429–1438.
105. Batool, S.; Liaqat, U.; Hussain, Z.; Sohail, M. Synthesis, characterization and process optimization of bone whitlockite. Nanomaterials **2020**, *10*, 1856.
106. Berzina-Cimdina, L.; Borodajenko, N. Research of calcium phosphates using Fourier transform infrared spectroscopy, Infrared spectroscopy-materials science, engineering and technology. **2012** *12*, 251–263.
107. Sa, Y.; Guo, Y.; Feng, X.; Wang, M.; Li, P.; Gao, Y.; Yang, X.; Jiang, T.; Are different crystallinity-index-calculating methods of hydroxyapatite efficient and consistent? New Journal of Chemistry **2017**, *41*, 5723–5731.
108. Theoret, A.; Sandorfy, C. Infrared spectra and crystalline phase transitions of ammonium nitrate. Canadian Journal of chemistry **1964**, *42*, 57–62.
109. Ahmed, S.; Ahsan, M. Synthesis of Ca-hydroxyapatite bioceramic from egg shell and its characterization. Bangladesh Journal of Scientific and Industrial Research **2008**, *43*, 501–512.
110. Kumar, G.S.; Thamizhavel, A.; Girija, E. Microwave conversion of eggshells into flower-like hydroxyapatite nanostructure for biomedical applications. Materials Letters **2012**, *76*, 198–200.

111. Ramesh, S.; Natasha, A.; Tan, C.; Bang, L.T.; Ching, C.; Chandran, H. Direct conversion of eggshell to hydroxyapatite ceramic by a sintering method. Ceramics international **2016**, *42*, 7824–7829.
112. Kamalanathan, P.; Ramesh, S.; Bang, L.; Niakan, A.; Tan, C.; Purbolaksono, J.; Chandran, H.; Teng, W. Synthesis and sintering of hydroxyapatite derived from eggshells as a calcium precursor. Ceramics International **2014**, *40*, 16349–16359.
113. Jang, H.L.; Jin, K.; Lee, J.; Kim, Y.; Nahm, S.H.; Hong, K.S.; Nam, K.T. Revisiting whitlockite, the second most abundant biomineral in bone: Nanocrystal synthesis in physiologically relevant conditions and biocompatibility evaluation. ACS nano **2014**, *8*, 634–641.
114. Diallo-Garcia, S.; Osman, M.B.; Krafft, J.-M.; Boujday, S.; Guylène, C. Discrimination of infrared fingerprints of bulk and surface POH and OH of hydroxyapatites. Catalysis Today **2014**, *226*, 81–88.
